# Effect of contextual diversity on word recognition in different semantic contexts

**DOI:** 10.1002/pchj.716

**Published:** 2023-12-17

**Authors:** Zhongchen Mu

**Affiliations:** ^1^ School of Psychology Nanjing Normal University Nanjing China

**Keywords:** contextual diversity, parafovea, semantic incongruence, word frequency, word recognition

## Abstract

Efficient word recognition is important to facilitate reading comprehension. Two important factors influence word recognition—word frequency (WF) and contextual diversity (CD)—but studies have not reached consistent conclusions on their role. Based on previous studies, the present study strictly controlled the anticipation of sentence context on target words. In the context of the semantic incongruence of Chinese sentences—that is, when the context is equivalent and low in anticipation of the target noun—CD effects were found on late processing indicators of the eye movement data of parafoveal words, and the CD feature of parafoveal words led to a significant parafoveal‐on‐foveal effect. However, none of these results were found in the semantically reasonable (semantic congruence) context. The results suggested that high CD words are better at adapting to unexposed or learned contexts, which was not the case for high WF words.

## INTRODUCTION

Reading comprehension is a basic skill in modern social life, and the ability to recognize words plays an important role in promoting reading comprehension. Word frequency (WF) and contextual diversity (CD) are two important factors in visual word recognition (Huang et al., 2020; Mendes et al., 2019). However, the findings on this topic have not been unanimous. WF has been defined as the number of occurrences of a word in a given corpus (Brysbaert et al., 2017). The processing time for high‐frequency words is shorter than that for low‐frequency words, and the accuracy rate is also higher for words with a lower frequency of occurrence (Forster & Chambers, 1973; Savin, 1963; Solomon & Howes, 1951).

However, Adelman et al. (2006) found that the time individuals spent on word processing in lexical decision and naming tasks was significantly influenced by CD rather than WF. CD refers to the number of documents in which a word appears in a corpus. Subsequent studies have also shown that CD, but not WF, influences the speed of individual word recognition (Cai & Brysbaert, 2010; Dimitropoulou et al., 2010; Perea et al., 2013; Soares et al., 2015). This finding is supported by research from behavioral studies (Chen et al., 2017; Huang et al., 2020; Plummer et al., 2014); however, neuro‐electrophysiological studies have reached a different conclusion, namely, that CD and WF in visual word recognition and reading are not substitutable for each other (Parmentier et al., 2017; Vergara‐Martínez et al., 2017).

Most previous studies have used lexical decision tasks (Cai & Brysbaert, 2010; Dimitropoulou et al., 2010; Perea et al., 2013; Soares et al., 2015), lexical naming tasks (Adelman et al., 2006), and natural reading (Chen et al., 2017; Plummer et al., 2014) to examine the effects of CD and WF on lexical recognition. Judgments (word or nonword) made by individuals in the lexical judgment task may be based on the representation of the word or activation of the word as a whole (a guess based on familiarity) (Grainger & Jacobs, 1996; Vergara‐Martínez & Swaab, 2012), whereas in a visual search task, only superficial comparisons of words are needed to ensure that the target word is identified. In addition, related studies have shown that, in lexical naming tasks, correctly naming words does not imply activation of lexical semantics (Shu et al., 2005). Rayner and Raney (1996) found that WF effects were only found in reading tasks and not in visual search tasks, perhaps because individuals need to process words for meaning (semantic extraction) to ensure lexical access in the reading task. Recent research on auditory word recognition has shown that the cohort entropy and phoneme surprisal effects are robust for continuous speech, but no cohort entropy effect has been observed when words are heard in isolation, suggesting that surprisal effects may require only wordform‐level activation, while cohort entropy effects may require lexical‐syntactic activation or higher. Similarly, phoneme surprisal effects could implicate up to lexical‐syntactic activation while cohort entropy effects require conceptual activation or higher (Gaston et al., 2023). Hence, lexical decision and naming tasks do not fully guarantee the activation of lexical semantics.

In light of the above, researchers have explored the effects of CD and WF on word recognition during normal reading (Chen et al., 2017; Huang et al., 2020; Plummer et al., 2014). The results suggest that CD is a more accurate predictor of word processing speed than WF within a normal reading task. However, these studies did not strictly control for additional variables (e.g., lexical category, lexical semantic diversity) (Plummer et al., 2014) and anticipation of target words (Chen et al., 2017). In an event‐related potential (ERP) study (Vergara‐Martínez et al., 2017), the characteristics of target words were, similarly, not well controlled; for example, there was a marginally significant difference in WF between high and low CD words. The above study made it possible to observe a mixed effect of CD and WF on word recognition, and a context conducive to high CD word recognition was created.

A high CD word indicates that the word appears in more and different contexts during the learning phase, so the likelihood of its appearing in any subsequent new context is higher (Adelman et al., 2006; Jones et al., 2012); it follows that high CD words adapted in unexposed or unlearned contexts may be relatively strong. Related research suggests that CD effects are more likely to be driven by contextual information, such as the topic (Steyvers & Griffiths, 2007) or the semantic features of the context (Adelman et al., 2006; Hoffman et al., 2012), and theories have also suggested that WF effects focus more on the order in which individuals detect feature symbols, such as the activation–verification model, which assumes that the degree of word activation depends on the degree of activation of the word's constituent letters in the alphabet (Paap et al., 1982). In contrast, explanations of CD also place greater emphasis on the integrated processing of words with the context or situation in which they are placed, in addition to their semantic processing. For example, the Bind‐Cue‐Decide Model of Episodic Memory (BCDMEM) emphasizes the extent to which the contextual memory of lexical activation matches the current lexicon (Dennis & Humphreys, 2001), and the dual processing model similarly emphasizes the importance of context for word recognition (Joordens & Hockley, 2000). This implies that the CD effect primarily reflects integrated context‐based processing, whereas the WF effect reflects form‐based perceptual processing (Vergara‐Martínez et al., 2017). In summary, the CD effect is more likely to be influenced by contextual conditions.

Chen et al. (2017) created structurally consistent contexts for the target nouns, but there was no control for the restrictiveness of the context in which the target words were placed or the expectation created by the context for the target words. Specifically, the context in which the high CD words were placed was highly restrictive; for example, the noun “antique” was more likely to appear after the verb “collect” than the nouns “meteorite” or “horn.” It created the context that is conducive to the recognition of high CD words, and which leads to the following questions: does the CD effect then persist when the verb in the sentence is matched with the target noun in a familiar and reasonable way (e.g., when the target word is expected to be equivalent)? If the effect is not significant or not present at all, is it more pronounced when the verb in the sentence is matched with the target noun in an unreasonable way (e.g., when the target word is expected to be equivalent and low), given that high CD words appear in more and different contexts and are more likely to appear in subsequent unknown contexts (Jones et al., 2012)? Furthermore, the influence of the CD and WF characteristics of parafoveal words on the recognition of foveal words needs to be further investigated. This study addresses these questions in two experiments that investigate the contextual conditions under which CD replaces WF as a better predictor of word recognition.

## EXPERIMENT 1: EFFECT OF CD ON WORD RECOGNITION IN SEMANTICALLY CONGRUENT CONTEXT

### Methods

#### Participants

A total of 40 participants (12 males), with an average age of 21.83 years, were recruited from a local university. The subjects had normal or corrected‐to‐normal vision; their native language was Chinese; all subjects were right‐handed. Informed consent was signed before the experiment, and the subjects were paid ¥45 on completion of the experiment.

#### Materials

Since there was no LH (Low WF − High CD) vocabulary (Chen et al., 2017; Plummer et al., 2014), 30 LWF group (Low WF − Low CD, LL), control group (High WF − Low CD, HL), and HCD group (High WF − High CD, HH) target words were selected from the SUBTLEX‐CH‐WF corpus based on the log values (for both WF and CD) (Cai & Brysbaert, 2010), all of which were two‐character inanimate nouns. There was no significant difference in WF between the HCD group and control group words, *t*(58) = 1.290, *p* = .202, and the HCD group had significantly higher CD than the control group words *t*(58) = 10.361, *p* < .001, *d* = 2.681, 95% CI = [0.316, 0.467]. There was no significant difference in CD between the LWF group and control group words, *t*(58) = −0.642, *p* = .523, and WF was significantly lower in the LWF group than in the control group *t*(78) = −18.615, *p* < .001, *d* = −1.957, 95% CI = [−0.493, −0.396]. Chinese sentences with anticipatory equivalence were created for each target word (Zhang et al., 2014), such as

何艳/缴纳了/那几笔/*罚款/*以便取走/被扣的货车.

He yan/ paid /**those**/ *fines* /to pick up/ the impounded truck

(The bolded words in the examples are foveal words and the italicized words are parafoveal words; these were presented in normal form in both the exercises and the formal experiments.)

The following variables were rated and controlled in this study to control additional variables. The target words (HCD group vs. control group, LWF group vs. control group) were evaluated separately for the WF of the first word (*t*s <0.205, *p*s > .838), CD (*t*s <0.724, *p*s > .472), and orthography (*t*s <1.203, *p*s > .234) and the WF of the last word (*t*s <1.461, *p*s > .150), CD (*t*s <1.028, *p*s > .308), and orthography (*t*s <0.556, *p*s > .580). The overall lexical strokes (*t*s < −0.978, *p*s > .332), semantics (*t*s <1.433, *p*s > .157), familiarity (*t*s < −0.603, *p*s > .549), and abstractness (*t*s <0.095, *p*s > .924) did not significantly differ. Values for each condition are presented in Appendix Table A1.

This experiment selected verbs that frequently match with the target words when creating sentences, but verbs are difficult to control in terms of features such as orthography, so quantifiers (e.g., a few, several) were inserted between the verbs and target nouns. The present study thus also rated the above factors for the quantifier located before the target words, and the quantifiers in the sentences containing the target words (HCD group vs. control group, LWF group vs. control group) were rated for the WF of the first character (*t*s <0.001, *p*s > .989), CD (*t*s <0.01, *p*s > .989), and orthography (*t*s <1.203, *p*s > .234); and the WF of second characters (*t*s <1.357, *p*s > .185), CD (*t*s <0.209, *p*s > .835), orthography (*t*s < −0.519, *p*s > .562); the overall word strokes (*t*s <0.281, *p*s > .160), semantics (all 1), familiarity (*t*s <1.354, *p*s > .181), and abstractness (*t*s <0.542, *p*s > .590) did not significantly differ. Values for each condition are presented in Appendix Table A2. Sentential constraint was defined as the cloze probability of the sentence continuation given with the highest probability across participants (Federmeier et al., 2010). However, the noun with the highest number of fillings in the sentence may not be the target noun; therefore, the contextual constraint of the sentence and the anticipation of the target word were assessed. The parts of each sentence from the beginning to the target noun (but not including it) were extracted, such as “He Yan paid those,” and participants were asked to fill in the first noun that came to mind. The percentage of nouns filled in the most was used as a measure of the contextual constraint of that sentence, and the percentage of target nouns filled in was calculated as a measure of the expectancy of that target noun (Li et al., 2015). A total of 50 subjects participated in this assessment, and the results showed that the means of the contextual restriction for sentences containing the HCD group and control group target words were 0.512 (SD = 0.124) and 0.505 (SD = 0.118), respectively, which were not significantly different: *t*(29) = 0.148, *p* = .883; the means of contextual restriction for sentences containing the control group and LWF group target words were 0.505 (SD = 0.118) and 0.508 (SD = 0.122), respectively, which were not significantly different: *t*(29) = 0.089, *p* = .930. The expectedness means of the HCD group and control group target words were 0.261 (SD = 0.13) and 0.259 (SD = 0.14), respectively, which were not significantly different: *t*(29) = 0.106, *p* = .916; the expectedness means of the control group and LWF group target words were 0.259 (SD = 0.14) and 0.254 (SD = 0.11), respectively, with a non‐significant difference: *t*(29) = 0.474, *p* = .639.

#### Procedure

**FIGURE 1:**
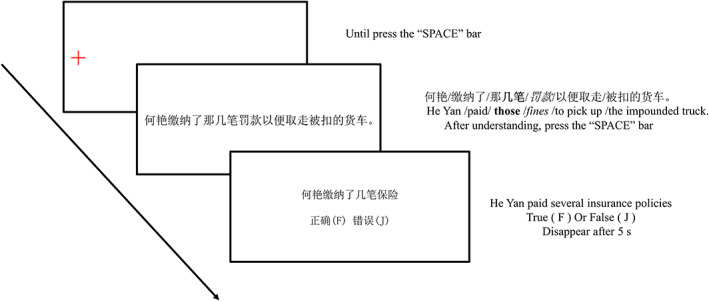
Trial scheme for Experiment 1.

During the experiment, only one participant at a time was allowed to take part. After participants arrived at the laboratory, they were first briefly introduced to the laboratory apparatus and environment. Subsequently, participants were presented with the instructions for the experimental procedure and asked to read them carefully. The experimental procedure was then introduced and explained to the participant to relieve any potential nervousness or excitement, and the questions raised were answered in detail to ensure that the participant accurately understood the experimental procedure and participated in the subsequent formal experiments in a relatively calm emotional state. After completing the exercise twice for calibration, participants formally entered the experiment and read the instructions again to enter the practice phase. There were 10 sentences in the practice block, and after reading and answering the questions as required, participants entered the formal experiment. The flow of the practice and formal experiment is shown in Figure 1. First, the red cross‐gaze point is presented, and the cross‐gaze point is aligned with the first word of the next screen of sentences. The participant pressed the space bar, and the red cross disappeared. Then, sentences appeared in the center of the screen. Participants were asked to read according to their usual manner and speed and to try to keep their head and body stable. The sentence disappeared by pressing the SPACE bar after participants understood; then, a true or false judgment question was presented and participants were asked to make a judgment based on the sentences they have just read. As in previous studies (Gaston et al., 2023), good performance on the task requires lexical‐syntactic and conceptual information access to have occurred for most stimuli because the probe words were unpredictable. Thus, the question in our study will change the following areas of the original sentence: (1) the animate noun, (2) the verb, (3) the quantity word, (4) the target noun, and (5) the rest of the sentence. To ensure the subjects' conscientiousness, as long as one of the questions presented did not match the original sentence, it was considered wrong. The experiment was divided into two blocks, containing three types of sentences presented immediately between the blocks. Participants were allowed to take a short break while answering the questions and between blocks, and pressed the SPACE bar after the break to continue the experiment. The procedure of this and the following experiments received ethical approval from the ethics review board of Nanjing Normal University, and written informed consent was obtained from the participants.

#### Eye movement data recording and analysis

Eye movement data were recorded using a Senso Motoric Instruments (SMIs) iView Hi‐Speed oculograph with a sampling rate of 1250 Hz. The distance of the monitor from the subject was 70 cm in a straight line, and the angle of view was approximately 1.22 degrees for each Chinese character. A 13‐point random calibration was performed using the WINCAR program for focusing, and the subject was calibrated twice in a row with an accuracy of fewer than 0.5 degrees in both X and Y directions before continuing the test. The test program used E‐Prime 2.0 to present the stimuli, and the experimental stimulus pictures and eye movement data were matched online by marking the eye movement data stream with the rem command.

Statistical analyses of the eye movement data were performed using first‐fixation duration (*FFD*), gaze duration (*GZD*), and skipping rate (*Skip*), which reflect early processing indicators, and regression rate (*RegIn*), which reflects late processing indicators, as well as Go‐past time (*Go‐past*) and total reading time (*TTime*), which reflect late processing indicators.

In this study, the data were analyzed using a linear mixed model (LMM) based on the R language (R Development Core Team, 2014) environment, and the lme4 data processing package (Bates et al., 2015) was used to analyze the data. Subjects and items were specified as crossover random effects when analyzing the data using LMM data processing techniques. Markov‐Chain Monte Carlo algorithms were used to derive post hoc distributed model parameters as estimates of significance, reflecting variation from both subjects and items (Baayen et al., 2008). Thus, the regression model included fixed effects (e.g., word type) and the maximal random effects structure with by‐participants and by‐items random intercepts and slopes (Barr et al., 2013; Chen et al., 2017; Huang et al., 2020; Matuschek et al., 2017). The complete model was LMM.RT = lmer (RT ~ type + (1 + type | subject) + (1 | item), data = data). The model was run with log transformations for the analyzed metrics and logistic lme transformations for the skipped and reread data. The linear model in this experiment was analyzed with lexical type as a fixed factor.

### Results

Following previous studies (Rayner, 1998, 2009), gaze points shorter than 80 ms or longer than 800 ms were removed, and data that did not meet the requirements were removed according to the following criteria: (1) less than four gazes on the sentence; (2) data with failed tracking (due to factors such as subject blinking, head movement, etc.); and (3) data with a mean located outside of three standard deviations. The deleted data accounted for 2.16% of the total data collected. The mean percentage of subjects' correct responses to the questions was 95.6%, indicating careful completion of the experimental task. The eye movement measures for foveal and parafoveal words were analyzed, and the results are shown in Tables 1 and 2. Comparisons of the control group and the LWF group assessed character frequency effects with CD controlled, and comparisons of the HCD group and the control group assessed character contextual diversity effects with WF controlled.

**TABLE 1:** Eye movement measures on foveal and parafoveal words in each group.

Target words	Analyzed indicators	HCD group	Control group	LWF group
Foveal words (The quantity word)	*FFD*	210 (84)	205 (89)	204 (81)
	*GZD*	245 (101)	233 (105)	231 (115)
	*Skip*	0.15 (0.35)	0.16 (0.36)	0.14 (0.15)
	*Go‐past*	245 (101)	242 (106)	240 (114)
	*RegIn*	0.16 (0.37)	0.15 (0.37)	0.14 (0.35)
	*TTime*	327 (185)	321 (169)	318 (152)
Parafoveal words (The target noun)	*FFD*	279 (84)	277 (82)	281 (80)
	*GZD*	350 (119)	355 (118)	358 (123)
	*Skip*	0.08 (0.27)	0.10 (0.29)	0.09 (0.28)
	*Go‐past*	448 (98)	453 (101)	457 (105)
	*RegIn*	0.26 (0.47)	0.28 (0.48)	0.29 (0.49)
	*TTime*	516 (216)	527 (207)	529 (213)

**TABLE 2:** Regression coefficients and test statistics from linear mixed effects for eye movement measures on the foveal and parafoveal words.

Target words	Analyzed indicators	Lexical type
Foveal words (The quantity word)	*FFD*	−0.026
	*GZD*	−0.031^§^
	*Skip*	−0.009
	*Go‐past*	−0.028
	*RegIn*	−0.024
	*TTime*	−0.020
Parafoveal words (The target noun)	*FFD*	0.009
	*GZD*	0.021
	*Skip*	0.002
	*Go‐past*	0.016
	*RegIn*	0.018
	*TTime*	0.024

§ *p* < .1.

The results of the eye movement measures for the foveal words indicated that none of the main effects of lexical type were significant (*FFD*: *b* = −0.026, SE = 0.018, *t* = −1.42, *p* = .160; *Skip*: *b* = −0.009, SE = 0.028, *t* = −0.33, *p* = .744; *Go‐past*: *b* = −0.028, SE = 0.018, *t* = −1.61, *p* = .112; *RegIn*: *b* = −0.024, SE = 0.022, *t* = −1.09, *p* = .277; *TTime*: *b* = −0.020, SE = 0.017, *t* = −1.15, *p* = .251); however, the main effect of lexical type reached marginal significance on *GZD*: *b* = −0.031, SE = 0.017, *t* = −1.65, *p* = .091, and post hoc comparisons revealed that foveal words located in front of HCD group words provoked longer gaze duration than foveal words located in front of control group and LWF group words (*p*_1_ = .098, *p*_2_ = .098), with no difference in gaze duration between foveal words located in front of control group and LWF group words (*p* = .822). None of the main effects of lexical type were significant in the results of the eye movement measures for parafoveal words (*FFD*: *b* = 0.009, SE = 0.017, *t* = 0.50, *p* = .618; *GZD*: *b* = 0.021, SE = 0.017, *t* = 1.23, *p* = .218; *Skip*: *b* = 0.002, SE = 0.034, *t* = 0.15, *p* = .882; *Go‐past*: *b* = 0.016, SE = 0.018, *t* = 0.91, *p* = .364; *RegIn*: *b* = 0.018, SE = 0.026, *t* = 0.94, *p* = .351; *TTime*: *b* = 0.024, SE = 0.018, *t* = 1.03, *p* = .198). In addition, the processing time (ms) of the whole sentence was analyzed and indicated that the main effect of lexical type was not significant (*b* = 0.007, SE = 0.022, *t* = 0.33, *p* = .764). The reading time (ms) of sentences containing HCD group words (*M* = 7068, SD = 1023) was lower than that of sentences containing control group words (*M* = 7087, SD = 1023) and LWF group words (*M* = 7162, SD = 1188), but the difference is not significant (all *p*s > .3115).

In sum, when the verb in the sentence matches the target noun in a familiar and reasonable way, similar contextual restrictiveness and target word anticipation weaken the recognition advantage of high CD words, and the results also showed no WF effect. In Experiment 2, the collocation of the verb with the target vocabulary was varied in each sentence, thus creating an experimental condition of semantic incongruence, which ensured that the target words were in a context to which subjects had not been exposed in the previous learning stage, and which also made the sentence context have the same and lower expectation for the target word. In this way, Experiment 2 explored the specific prerequisite (background) conditions that would provide an advantage for high CD words.

## EXPERIMENT 2: THE EFFECT OF CD ON WORD RECOGNITION IN A SEMANTICALLY INCONGRUENT CONTEXT

### Methods

#### Participants

Forty‐two participants (15 male), with an average age of 22.05 years, were recruited from a local university. The subjects had normal or corrected‐to‐normal vision, their native language was Chinese, and all were right‐handed. Informed consent was signed before the experiment, and the subjects were paid ¥45 on completion of the experiment.

#### Materials

The selection and evaluation of target nouns and quantity words were the same as in Experiment 1. The semantic relationship between the verb and the target noun was changed in each sentence to form an experimental condition of semantic incongruence (the bolded words in the examples are foveal words and the italicized words are parafoveal words; these were presented in normal form in both the exercises and the formal experiments).

Semantic congruence: He Yan /paid /**several** /*fines/* to take away/ the impounded truck.

何艳/缴纳了/那几笔/*罚款/*以便取走/被扣的货车.

Semantic incongruence: He Yan/ piled up /**several**/ *fines* /to take away/ the impounded truck.

何艳/堆积了/那几笔/*罚款/*以便取走/被扣的货车.

The contextual restriction and the expectedness of the target words were evaluated, and the percentage of subjects who filled in the most common noun was used as an indicator of the contextual restriction of the sentence, and the percentage of subjects who filled in the target noun was calculated as an indicator of the expectancy of the target noun (Li et al., 2015). A total of 49 subjects participated in this evaluation. The results showed that the means of the contextual restriction in the semantic congruence and incongruence conditions were 0.52 (SD = 0.13) and 0.51 (SD = 0.12), respectively, which were not significantly different: *t*(89) = 0.562, *p* = .576. The means of target noun expectancy in the semantic congruence and incongruence conditions were 0.28 (SD = 0.23) and 0, respectively, which were significantly different: *t*(89) = 11.201, *p* < .001.

In addition, to ensure the validity of the whole‐sentence and local contextual semantic reasonableness manipulations of the key nouns, the whole‐sentence and half‐sentence semantic reasonableness ratings were conducted separately for the experimental sentences in this study. In the half‐sentence reasonableness rating, the sentence material was presented up to and including key nouns (e.g., “He Yan paid several fines”). The subjects were asked to read the sentence carefully and rate the semantic plausibility of the sentence on a 5‐point Likert scale, where 1 is *very unreasonable* and 5 is *very reasonable*. The results of the two assessments, in which 32 subjects each participated, showed that the mean scores for the whole‐sentence reasonableness assessment were 4.32 (SD = 0.39) and 1.54 (SD = 0.41) for the semantic congruence and incongruence conditions, respectively, with a significant difference: *t*(89) = 49.233, *p* < .001. For the half‐sentence reasonableness assessment, the mean scores for the semantic congruence and incongruence condition were 4.28 (SD = 0.38) and 1.42 (SD = 0.34), respectively, with also a significant difference: *t*(89) = 62.171, *p* < .001. In addition, in both whole‐ and half‐sentence reasonableness ratings under the semantic incongruence condition, the differences in reasonableness between the sentences containing HCD group and control group words or control group and LWF group words were not mutually significant (all *t*s <0.832, all *p*s > .784).

#### Procedure and eye movement data recording and analysis

The same procedure for eye movement data recording and analysis was followed as in Experiment 1.

### Results

Following previous studies (Rayner, 1998, 2009), gaze points shorter than 80 ms or longer than 800 ms were removed, and data that did not meet the requirements were removed according to the following criteria: (1) fewer than four gazes on the sentence; (2) data with failed tracking (due to factors such as subject blinking, head movement, etc.); and (3) data with a mean located outside of three standard deviations. The deleted data accounted for 2.21% of the total data collected. The mean percentage of participants' correct responses to the questions was 94.2%, indicating careful completion of the experimental task. The eye movement measures for foveal and parafoveal words were analyzed, and the results are shown in Tables 3 and 4.

**TABLE 3:** Eye movement measures on foveal and parafoveal words in each group.

Target words	Analyzed indicators	HCD group	Control group	LWF group
Foveal words (The quantity word)	*FFD*	204 (88)	193 (81)	191 (78)
	*GZD*	233 (103)	222 (108)	220 (101)
	*Skip*	0.16 (0.37)	0.18 (0.38)	0.19 (0.39)
	*Go‐past*	235 (106)	223 (106)	220 (101)
	*RegIn*	0.31 (0.46)	0.30 (0.46)	0.30 (0.45)
	*TTime*	432 (192)	429 (198)	424 (191)
Parafoveal words (The target noun)	*FFD*	294 (82)	292 (84)	297 (84)
	*GZD*	359 (104)	357 (112)	355 (105)
	*Skip*	0.07 (0.26)	0.08 (0.27)	0.08 (0.27)
	*Go‐past*	533 (111)	544 (109)	547 (107)
	*RegIn*	0.49 (0.63)	0.55 (0.62)	0.54 (0.62)
	*TTime*	649 (194)	673 (208)	681 (220)

**TABLE 4:** Regression coefficients and test statistics from linear mixed effects for eye movement measures on the foveal and parafoveal words.

Target words	Analyzed indicators	Lexical type
Foveal words (The quantity word)	*FFD*	−0.054**
	*GZD*	−0.049**
	*Skip*	0.013
	*Go‐past*	−0.049**
	*RegIn*	−0.016
	*TTime*	−0.010
Parafoveal words (The target noun)	*FFD*	0.018
	*GZD*	−0.016
	*Skip*	0.012
	*Go‐past*	0.053**
	*RegIn*	0.058*
	*TTime*	0.08***

*** *p* < .001;

** *p* < .01;

* *p* < .05.

The results of the eye movement measures for the foveal words indicated that the main effects of lexical type on *Skip* (*b* = 0.013, SE = 0.027, *t* = 1.01, *p* = .317), *RegIn* (*b* = −0.016, SE = 0.026, *t* = −0.66, *p* = .513), and *TTime* (*b* = −0.010, SE = 0.018, *t* = −0.85, *p* = .400) were not significant. The main effect of lexical type was significant on *FFD* (*b* = −0.054, SE = 0.017, *t* = −3.14, *p* < .01), *GZD* (*b* = −0.049, SE = 0.017, *t* = −2.86, *p* < .01), and *Go‐past* (*b* = −0.049, SE = 0.017, *t* = −2.86, *p* < .01). The foveal words located in front of HCD group words showed significantly larger effects than those located in front of control group and LWF group words in terms of *FFD* (*p*_1_ < .05, *p*_2_ < .01), *GZD* (*p*_1_ < .05, *p*_2_ < .05), and *Go‐past* (*p*_1_ < .05, *p*_2_ < .05). The foveal words located in front of control group words were not significantly different from those in front of LWF group words in terms of *FFD*, *GZD*, and *Go‐past* (all *p*s > .826).

The results of the eye movement measures for the parafoveal words showed that the main effects of lexical type on *FFD* (*b* = 0.018, SE = 0.020, *t* = 0.93, *p* = .361), *GZD* (*b* = −0.016, SE = 0.017, *t* = −0.96, *p* = .335), and *Skip* (*b* = 0.012, SE = 0.032, *t* = 0.38, *p* = .708) were not significant. The main effects of lexical type on *Go‐past* (*b* = 0.053, SE = 0.017, *t* = 3.13, *p* < .01), *RegIn* (*b* = 0.058, SE = 0.024, *t* = 2.39, *p* < .05), and *TTime* (*b* = 0.080, SE = 0.017, *t* = 4.63, *p* < 0.001) were significant. The effects of parafoveal HCD group words were significantly smaller than the parafoveal control group and LWF group words in terms of *Go‐past* (*p*_1_ < .05, *p*_2_ < .01), *RegIn* (*p*_1_ < .05, *p*_2_ < .05), and *TTime* (*p*_1_ < .05, *p*_2_ < 0.001). Parafoveal control group words did not differ significantly from parafoveal LWF group words in *FFD*, *GZD*, and *Skip* (all *p*s > .2943). Similarly, the processing time of the whole sentence was analyzed and it was found that the main effect of lexical type was significant (*b* = 0.184, SE = 0.020, *t* = 9.20, *p* < 0.001). The reading time (ms) of sentences containing HCD group words (*M* = 8457, SD = 1032) was significantly lower than that of sentences containing control group words (*M* = 8840, SD = 1012) and LWF group words (*M* = 8923, SD = 1008) (all *p*s < .001), and, there was no difference in reading time between sentences containing control group words (*M* = 8840, SD = 1012) and sentences containing LWF group words (*M* = 8923, SD = 1008) (*p* > .156).

Finally, this study observed a main CD effect in the incongruent context. The high CD words are more able to show their advantageous features when in the incongruent context, i.e., their advantage may be better reflected in an unexposed (or unlearned) context, or the high CD words are more able to adapt to new contexts, but the high WF words do not have this ability.

## DISCUSSION

This study focused on the prerequisites of the CD features of words to facilitate word recognition and integration during sentence reading. The results of Experiment 1 showed that a CD effect trend was found on eye movement indicators for early and late processing of parafoveal words in a Chinese semantically reasonable sentence—that is, when the context has the same expectedness for the target noun—but the results were not significant. When the foveal words were simple quantity words (e.g., a few, several), the CD feature of the parafoveal words led to an insignificant parafoveal‐on‐foveal effect. The above results suggest that semantically plausible contexts weaken the recognition advantage of high CD words. However, the results of Experiment 2 showed that CD effects arise on late processing indicators of parafoveal words when the sentence context was incongruent, and the CD feature of parafoveal words led to a significant parafoveal‐on‐foveal effect.

Compared to previous studies (in which the target nouns appeared after a verb with which they were infrequently collocated), target nouns in the present experiment appeared after a verb with which they were frequently collocated, making the context more predictable for target nouns, thus, higher contextual restrictions or expectations made individuals attend to the target word for a shorter period. Murray and Rowan (1998) found that subject‐verb collocation plausibility (e.g., uranium smacked the child—savages smacked the child) affected individuals' processing time for the subject and the sentence as a whole. Becker (1979) used word pairs with different degrees of semantic relevance (e.g., semantically strongly relevant word pairs: FREEZING–COLD; semantically moderately relevant word pairs: REASON–MOTIVE; and semantically irrelevant word pairs: DRINK–MYTH) to explore the role of semantic context and WF effect in lexical recognition. Becker found that semantic context contributed significantly more to the recognition speed of low‐frequency words than high‐frequency words. This shows that higher restriction, anticipation, and reasonableness of the context accelerate the recognition of target words, especially for low CD or low WF words, so the difference in recognition time of the three types of target words (HCD group, control group, and LWF group) is greatly reduced. This may therefore make the identification and integration advantages of high CD words less significant.

When the foveal words in this study were simple quantity words (e.g., a few strokes, a few, or a few pieces), this resulted in a significant reduction in the cognitive load of the foveal words, which is consistent with the findings of Kennedy et al. (2002) that when the foveal words (gaze words) were short and the right non‐gaze words were long, low‐frequency words with uncommon first few letters, the gaze time on the foveal words decreased—that is, a reverse parafoveal‐on‐foveal effect occurs. Thus, the low load and higher skip rate of the foveal words may have resulted in sufficient and nearly equal attentional resources for all three types of parafoveal words; moreover, the higher restriction, anticipation, and reasonableness of the context contributed significantly to the low CD or low WF words, reducing the difference in the cognitive conflict caused by the three types of target words in the parafoveal position. The smaller cognitive conflict differences for the parafoveal words and the sufficient attentional resources may make the CD features of the parafoveal words lead to a less significant reverse parafoveal‐on‐foveal effect.

Related studies have shown that late processing indicators (e.g., go‐past time and regression rate) can reflect the processing difficulties encountered by readers in reading target regions (Pickering & Frisson, 2001) and the difficulty of semantic information access, as well as integration (Juhasz & Rayner, 2003). It can be seen that high CD parafoveal words are less difficult to integrate into the semantic incongruence condition than low CD words, but high and low WF parafoveal words do not differ as described above. The different levels of difficulty in integrating parafoveal words into the current context cause three types of target words to require unequal amounts of attentional resources, thus eliciting a reverse parafoveal‐on‐foveal effect. Studies have also shown that target words in the verb–object pairing implausible condition induce a greater N400 component than keywords in semantically violated conditions (Zhang et al., 2019). Thus, compared with parafoveal control group and LWF group words, parafoveal HCD group words are less difficult to integrate into the current context in the semantic incongruence condition, possibly because they induce relatively less cognitive conflict, making the parafoveal position require fewer attentional resources, thus producing a reverse parafoveal‐on‐foveal effect. Related studies have shown that high CD words imply that words appear in more and different contexts and are more likely to appear in subsequent unknown contexts (Jones et al., 2012). This perhaps suggests that high CD words may have a greater ability to adapt to contexts to which the reader was previously unexposed. Therefore, in semantic incongruence sentences, this study used verb–object collocation implausibility as an unexposed context, and the results are consistent with our expectations that high CD words have an integration advantage in semantic incongruence contexts compared to low CD words, or that high CD words can be more easily (and quickly) integrated into semantic incongruence contexts, which is an ability that high WF words do not have.

### Limitations and future directions

There are some limitations to this study. First, in the context created for the target words in this study, foveal words were simple quantity words (e.g., a few strokes, a few, or a few pieces), which resulted in a much lower cognitive load on the foveal words and a high skip reading rate. Further research should explore the effect of the CD and WF features of parafoveal words on the recognition of the foveal words and the parafoveal word itself under different foveal load conditions. Second, the present study found that high CD words can integrate quickly into semantic incongruence contexts, but the mechanism by which this occurs is not clear. Barber et al. (2012) and Li et al. (2015) created semantically reasonable and semantically violated experimental conditions by changing the collocation relationship between nouns and verbs (e.g., “Mary bought her new bike/head last week”). They found that after the critical screen (her new bike) was presented, the semantic incongruence (her new head) condition induced a larger N400 component compared to the semantically reasonable condition. Combined with the findings of this study, this suggests that high CD words may produce less cognitive conflict than low CD words in the parafoveal position in the semantic incongruence context, and a direct test of this hypothesis should be performed in the future. Finally, the full range of WF and CD features should be used to avoid selecting unusual words and keeping the number of stimuli rather small which reduces the effect sizes that can be investigated with good power, and in this study, the overall sentence reading speed (approximately 114–145 words per minute) was slower than the individual reading speed (238 words per minute) in a related study (Brysbaert, 2019). Due to the sentence judgment task (e.g., reading sentences repeatedly before answering questions), it is necessary to control for the individual's reading speed, in order to exclude the differences in reading speed and habits of each person when using ERP technology to explore the conditions and mechanisms under which high CD words have an advantage in future studies.

## CONCLUSION

The current study showed that high CD words could be more rapidly integrated into semantic incongruence contexts, but high WF words did not present this ability, and CD could replace WF as a better estimator of visual word recognition in semantic incongruence contexts.

## CONFLICT OF INTEREST STATEMENT

The author declare that the research was conducted in the absence of any commercial or financial relationships that could be construed as a potential conflict of interest.

## ETHICS STATEMENT

All study participants provided written informed consent. The study design was approved by the Ethics Review Board of Nanjing Normal University.

## APPENDIX A

**TABLE A1:** Characteristics of the target words (Parafoveal word) in Experiment 1 and Experiment 2.

Measure	HCD group	Control group	LWF group
Mean word frequency (log)	2.15 (0.12)	2.10 (0.13)	1.65 (0.29)
Mean contextual diversity (log)	2.01 (0.12)	1.62 (0.17)	1.60 (0.13)
Word frequency range (log)	1.99–2.74	1.91–2.38	1.59–2.74
Contextual diversity range (log)	1.90–2.54	1.32–1.83	1.53–1.66
WF of the first character (log)	3.01 (1.00)	2.98 (0.81)	3.02 (0.77)
CD of the first character (log)	2.72 (1.00)	2.64 (0.77)	2.78 (0.67)
WF of second characters (log)	3.09 (0.88)	2.69 (1.04)	2.84 (0.77)
CD of second characters (log)	2.79 (0.75)	2.43 (0.92)	2.64 (0.70)
Strokes of the first character	9.13 (2.71)	8.43 (2.94)	8.47 (3.10)
Strokes of the second character	8.01 (3.49)	9.82 (4.08)	7.93 (3.71)
Overall lexical strokes	17.14 (4.13)	17.71 (5.30)	16.40 (4.82)
Semantics	1.37 (0.61)	1.13 (0.43)	1.30 (0.47)
Orthography of the first character	400.16 (396.99)	217.26 (284.54)	325.27 (401.02)
Orthography of the second character	243.60 (314.05)	251.07 (324.85)	301.33 (373.71)
Orthography	321.83 (263.35)	234.167 (222.22)	313.30 (288.55)
Abstractness	5.12 (0.63)	5.21 (0.49)	5.17 (0.52)
Familiarity	5.93 (0.60)	5.92 (0.61)	5.94 (0.62)

**TABLE A2:** Characteristics of the quantity words (foveal word) in Experiment 1 and Experiment 2.

Measure	HCD group	Control group	LWF group
WF of the first character (log)	4.35 (0)	4.35 (0)	4.35 (0)
CD of the first character (log)	3.74 (0)	3.74 (0)	3.74 (0)
WF of second characters (log)	4.23 (0.72)	4.26 (0.78)	4.62 (0.71)
CD of second characters (log)	3.54 (0.26)	3.22 (0.32)	3.66 (0.19)
Strokes of the first character	2 (0)	2 (0)	2 (0)
Strokes of the second character	6.23 (2.69)	6.03 (2.82)	5.03 (2.62)
Overall lexical strokes	8.23 (2.69)	8.03 (2.82)	7.03 (2.62)
Semantics	1 (0)	1 (0)	1 (0)
Orthography of the first character	20 (0)	20 (0)	20 (0)
Orthography of the second character	268.07 (291.45)	314.07 (388.29)	258.17 (354.23)
Orthography	144.03 (145.73)	167.03 (194.15)	139.08 (177.11)
Abstractness	6.56 (0.30)	6.52 (0.32)	6.53 (0.30)
Familiarity	6.45 (0.30)	6.41 (0.28)	6.51 (0.30)
